# Development and validation of a measure of early adverse experiences: childhood adversity scale

**DOI:** 10.3389/fpsyt.2026.1701294

**Published:** 2026-05-12

**Authors:** Medaim Yanik, Burcu Uysal, Seyda Eruyar, Ayse Nur Dogan Ceyhan, Erkan Hasan Atalmış

**Affiliations:** 1 Department of Psychology, Ibn Haldun University, İstanbul, Türkiye; 2 Educational Science Department, Boğaziçi University, İstanbul, Türkiye; 3 Department of Physical Education and Sports, Manisa Celal Bayar University, Manisa, Türkiye

**Keywords:** childhood adversity, scale, scale development, trauma assessment, validity and reliability

## Abstract

**Introduction:**

Childhood adversities disrupt the healthy development of children and often have long-term effects on their physical and mental health in adulthood. Identifying them is essential; however, the majority of the existing tools do not adequately capture their diversity and complexity. Moreover, although the age at which adversities occur and their subjective impacts are known to be important, many instruments fail to assess these dimensions.

**Methods:**

To address these gaps, a new scale was developed based on a review of 300 client files from individuals diagnosed with dissociative identity disorder (DID). The scale items were derived from types of childhood adversity commonly associated with the formation of alternate identities. Psychometric evaluation was conducted using data from three independent samples.

**Results:**

Exploratory factor analysis (EFA) with the first sample (*n* = 338) indicated that a single factor explained more than half of the total variance, supporting a unidimensional structure for the 59-item scale. Confirmatory factor analysis (CFA) with the second sample (*n* = 413), refined using modification indices, demonstrated good model fit. Convergent validity was evaluated in a third sample (*n* = 125) using the Adverse Childhood Experiences (ACE) scale. A significant positive correlation was found between the Childhood Adversity Scale (CAS) and ACE, indicating strong convergent validity.

**Discussion:**

This suggests that CAS is a reliable and valid tool that both clinicians and researchers can use for a comprehensive assessment of childhood adverseness.

## Introduction

There is no single definition of childhood adversity (1). In his early work, Felitti (2) suggested that adverse childhood experiences are not only limited to psychological, sexual, or emotional abuse but also involve family-related issues, such as living with a family member affected by substance misuse or mental health difficulties. Later, the World Health Organization’s International Questionnaire on Adverse Childhood Experiences (ACE-IQ; 3) included additional adversities such as bullying and parental death as examples of adverse childhood experiences. The present study adopts the conceptual framework of McLaughlin and Sheridan (4). They conceptualize childhood adversity as experiences that fall outside the normative developmental contexts and require substantial psychological, social, and neurodevelopmental adaptation. This approach encompasses factors such as abuse, domestic violence, natural disasters, and many environmental stressors that affect development.

A large body of research suggests that adverse childhood experiences have a negative impact on both physical and psychological wellbeing (5). There is substantial evidence that adverse childhood experiences are associated with a wide range of psychopathologies and risk behaviors, including post-traumatic stress disorder (PTSD), depression, anxiety disorders, suicidal ideation and attempts, smoking, excessive alcohol consumption, and substance abuse (6). Furthermore, cumulative exposure to these adverse experiences further increases the risk of negative mental health outcomes (7). Moreover, research has demonstrated that the type, timing, severity, and duration of adverse experiences also play a significant role in determining the nature and severity of subsequent psychopathologies and risk behaviors (8).

Reliable and valid measurement and evaluation are necessary to understand and prevent the long-term consequences of adverse childhood experiences. In this context, numerous instruments have been developed to measure adverse childhood experiences in previous studies. However, the majority of these tools have limitations in fully encompassing the full complexity of these experiences. For instance, the widely used Childhood Trauma Questionnaire (CTQ) (9) assesses physical, emotional, and sexual abuse, along with physical and emotional neglect, but does not include other important adversities such as exposure to domestic violence or natural disasters. Similarly, the Life Experience Questionnaire (LEQ) (10) focuses primarily on physical and sexual abuse and neglect, while other tools, such as the Sexual Abuse Exposure Questionnaire (SAEQ) (11), are limited to the assessment of a single type of trauma.

Considering the limitations of the existing scales in the field, there is a substantial need to develop comprehensive, sensitive, and culturally relevant measures. Because adverse childhood experiences are diverse, complex, and multidimensional, clinicians may easily fail to detect them without the use of such measures (12), which may result in difficulty in understanding the overall picture and in planning appropriate interventions (13). Furthermore, because many of the Adverse Childhood Experiences (ACE) instruments were developed with a Western cultural focus, they may not adequately reflect the sociocultural characteristics of Eastern populations, which can limit the cross-cultural applicability of the existing scales (14).

In this context, a newly developed, culturally oriented, multidimensional instrument with strong content validity can facilitate a more accurate assessment and inform clinical practice. Therefore, the aim of this study was to develop a reliable and valid Childhood Adversity Scale (CAS) to address these needs. The CAS to be developed differs from the existing scales in several ways. Firstly, it is designed as a Likert-type scale, going beyond simple presence–absence (yes/no) assessments. Secondly, incorporates multidimensional aspects such as the type of adversity, the developmental timing of the adversity, and the perceived psychological impact. Finally, it combines three key parameters: the exposure level, the age of exposure, and the perceived impact. These features can make the CAS valuable for both clinical assessment and research applications.

## Methods

This study used a quantitative approach to develop the CAS. Standard scale development procedures were followed throughout the process.

### Sampling

The participants in this study were adults aged 18 years and over living in Turkey. The participant sample was obtained using convenience sampling, a non-random sampling technique. In this method, participants are selected based on their availability, accessibility, and proximity to the researcher (15, 16). Before data collection, the sample size was determined using criteria such as the number of items or underlying factors. Comrey and Lee (17) proposed a rule of thumb for assessing sample size adequacy in factor analysis, categorizing it as follows: 100 = poor, 200 = adequate, 300 = good, 500 = very good, and 1000+ = excellent. Moreover, Bryman and Cramer (18) recommended that the sample size be at least 5–10 times the number of items in the factor analysis. In this study, exploratory factor analysis (EFA), confirmatory factor analysis (CFA), and convergent validity assessment were conducted to develop CAS. Hurley et al. (1997) (19) suggested that EFA and CFA should not be applied to the same data due to overfitting and inflated model fit. Therefore, three separate groups of adults were included as participants in this study. The first group (*n* = 338) was used for EFA, the second group (*n* = 413) for CFA, and the third group (*n* = 125) for convergent validity assessment. **Table 1** presents the descriptive statistics regarding the personal characteristics of each group.

**Table 1:** Descriptive statistics on the characteristics of the included adults.

	Group for EFA (n=338)	Group for CFA (n=413)	Group for convergent validity (n=125)
Age			
Mean ± SD	33.68 ± 9.65	33.06 ± 10.43	34.66 ± 9.63
Min-Max	18-62	18-83	18-58
Gender			
Male	45 (13.3%)	63 (15.3%)	21 (16.8%)
Female	293 (86.7%)	350 (84.7%)	104 (83.7%)
Marital Status			
Single	126 (37.3%)	154 (37.3%)	49 (39.2%)
Divorced	12 (3.6%)	18 (4.4%)	3 (2.4%)
Married	200 (59.2%)	241 (58.4%)	73 (58.4%)
Educational Level			
Elementary	18 (5.3%)	26 (6.3%)	6 (4.8%)
High School	74 (21.9%)	105 (25.4)	22 (17.6%)
Bachelor	192 (56.8%)	223 (54.0%)	75 (60.0)
Graduate	60 (16.0%)	59 (14.2%)	22 (17.6%)
Working Status			
Working	142 (42.0%)	170 (41.2%)	55 (44.0%)
Not working	196 (58.0%)	243 (58.8%)	70 (56.0%)
Income level			
Low	61 (18.0%)	81 (19.6%)	24 (19.2%)
Middle	255 (75.4%)	304 (73.6%)	71 (72.8%)
High	22 (6.5%)	28 (6.8%)	10 (8.0%)
Do you have any chronic illness?			
Yes	80 (23.7%)	94 (22.8%)	36 (28.8)
No	258 (76.3%)	319 (77.2%)	89 (71.2%)
Have you ever received psychological support before?			
Yes	90 (26.6%)	120 (29.1%)	30 (24.0%)
No	248 (73.4%)	293 (70.9%)	95 (76.0%)
Have you received psychiatric history before?			
Yes	133 (39.3%)	158 (38.3%)	55 (44.0%)
No	205 (60.7%)	255 (61.7%)	70 (56.0%)

Data were collected online through announcements within the researchers’ networks and in online platforms. Invitations were extended through therapy centers where the authors work to reach the clinical sample in the final two phases. The first phase was conducted in 2020, while the second and third phases were conducted in 2024 and 2025.

The sample characteristics are summarized in **Table 1**.

**Table 1** presents groups that were generally similar in age (mean of approximately 33–34 years) and gender distribution (approximately 84%–87% female). The majority of the participants in all three groups were married (around 58%–59%), held a bachelor’s degree (approximately 54%–60%), and reported middle-income levels (approximately 73%–75%).

Regarding health-related characteristics, the majority of the participants across all three groups (EFA, CFA, and convergent validity) reported no chronic illness (71.2%–77.2%) and had not received psychological support (70.9%–76.0%). However, a significant proportion of both groups reported a history of psychiatric treatment (ranging from 38.3% to 44.0%).

### Assessment measures

#### Childhood Adversity Scale

The development of the CAS items was conducted in several stages. MY, one of the authors of this study, specializes in the treatment of patients with dissociative identity disorder (DID) in his clinic. The item generation process utilized approximately 300 patient files from MY and involved five clinical psychologists in the clinic. Two independent researchers analyzed the contents of the clinical observations and the session notes for the DID patients in these files. A large number of trauma accounts were identified during the file review process. Recurrent traumatic experiences were analyzed thematically guided by McLaughlin and Sheridan’s (4) framework on childhood adverse experiences. Specifically, items were included if they represent experiences that deviate from normative developmental environments and have been shown in previous literature to meaningfully impact development. Based on these criteria, domains such as abuse, neglect, domestic dysfunction, and severe environmental stressors were retained. Thematic analysis with the aforementioned framework led to the identification of 59 potentially adverse events, such as “being locked in the closet.” The final version of the scale was developed based on expert feedback from two clinician–academics, both of whom are co-authors of this study. These traumatic experiences were subsequently included in MY’s book, titled Raising Children: Traumatic Mistakes and Nurturing Truths, which was published in 2019 (20). The CAS item pool was created based on the content of this book. The adverse experiences listed in **Appendix 2** of this manuscript are directly adapted from MY’s book.

The questions first inquire whether the individual experienced childhood adversity. For those who answered “yes,” the respondents are then asked to specify their age at the time and to rate the impact on a five-point Likert scale, where the responses range from 1 (strongly disagree) to 5 (completely agree). The data collection process involved voluntary participants, who were informed about the study and the data collection tool before participation.

The instructions for CAS are presented at the beginning of the scale as follows:

“The statements below describe possible adverse experiences that may have occurred during your childhood. If a statement applies to you, please select ‘Yes’. If it does not apply, select ‘No’. When you select ‘Yes’, additional follow-up questions will appear, asking about the frequency of the event, the age(s) at which it occurred, and the extent to which it affected you.”

The following is an example item from the scale.

When I was a child, my parents compared me to other children. This statement applies to me: Yes, No.

Participants who selected “Yes” were prompted to answer three follow-up questions.

1. How often did you experience this?

Seldom, Sometimes, Often, Very often, Always.

2. During which childhood period(s) did this event occur?

- Infancy (0–1 year).
- Toddlerhood (1–3 years).
- Preschool age (3–6 years).
- Early school age (6–9 years).
- Middle childhood (9–11 years).

3. How much did this event affect you?

Not at all, A little, Moderately, Very much, Extremely.

The original scale is provided in the **Appendix**.

#### Adverse childhood experiences questionnaire

The ACE questionnaire was developed by Felitti et al. (2). It comprises 10 items with dichotomous response options (1 = “yes” and 0 = “no”), indicating whether participants experienced each ACE before age 18. It includes items on abuse (emotional, physical, and sexual), neglect (emotional and physical), and household dysfunction (such as parental separation/divorce and substance use).

### Data analysis

The suitability of the data for factor analysis was evaluated conducting the Kaiser–Meyer–Olkin (KMO) coefficient and Bartlett’s sphericity test in SPSS 22.0. Field (21) suggests that the Bartlett’s sphericity test must be significant and the KMO value must exceed 0.50 for the EFA to be appropriate. He further categorizes the KMO values as follows: 0.50–0.70 as “mediocre,” 0.70–0.80 as “good,” 0.80–0.90 as “excellent,” and values above 0.90 as “higher.” To perform CFA, MPlus 7.4 was used, and model fit indices, including the comparative fit index (CFI), the Tucker–Lewis index (TLI), root mean square error of approximation (RMSEA), and standardized root mean square residual (SRMR), were calculated. According to Kline (22), a model is considered acceptable if the CFI and TLI values are greater than 0.90 and the RMSEA and SRMR values remain below 0.08. For the convergent validity of the CAS, the ACE questionnaire (2) was used. To determine convergent validity, Pearson’s correlation coefficient was calculated between the CAS and the ACE. In this study, the Cronbach’s *α* of ACE was 0.77. According to Cohen (23) and Cohen (24), correlation coefficients of 0.50 or higher indicate large magnitude.

## Results

### Exploratory factor analysis

After calculation of the KMO coefficient and Bartlett’s sphericity test in SPSS 22.0, the findings showed a KMO of 0.97, indicating that the dataset is suitable for EFA. According to Field (21), a KMO value above 0.90 is considered excellent, suggesting that the correlations between variables are sufficient for factor extraction. In addition, Bartlett’s sphericity test was significant (*χ*^2^ = 36,590.81, *df* = 1,711, *p* < 0.01), confirming that the correlation matrix is not an identity matrix (25). This indicates that there are meaningful relationships among the variables, further justifying the use of EFA to identify underlying factor structures in the dataset. In light of these results, it was determined that the data were suitable for factor analysis.

**Figure 1:**
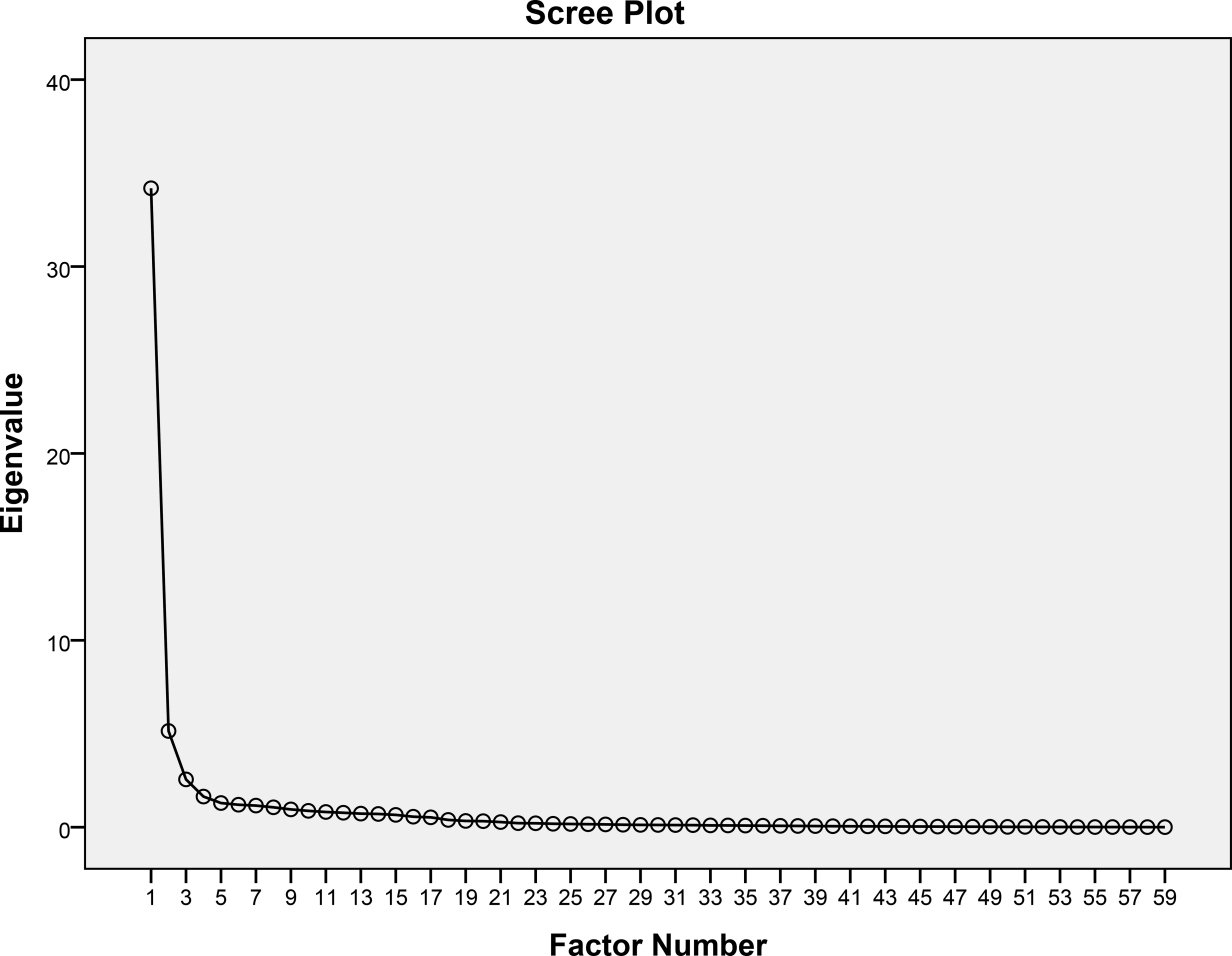
Scree plot.

Principal axis factoring (PAF) was conducted based on the criteria of the scree plot (see **Figure 1**) and an eigenvalue >1.0, which was found to account for 81.79% of the total variance in the scale. PAF, an EFA method, is preferred over principal component analysis as it focuses only on common variance, which is important for theory development (26).

Examination of the eigenvalues showed eight factors with eigenvalues greater than 1. Out of all factors, 57.95% of the variance was explained by factor 1, with 34.19, and 8.73% of the variance was explained by factor 2, with 5.15. Considering the eigenvalue and the explained variance, factor 1 was found to be approximately six times more dominant than factor 2. Moreover, factor 1 explained more than 50% of the total variance. Therefore, the scale was considered a single dimension (27, 28) and no rotation was applied (29).

### Confirmatory factor analysis and reliability

**Figure 2:**
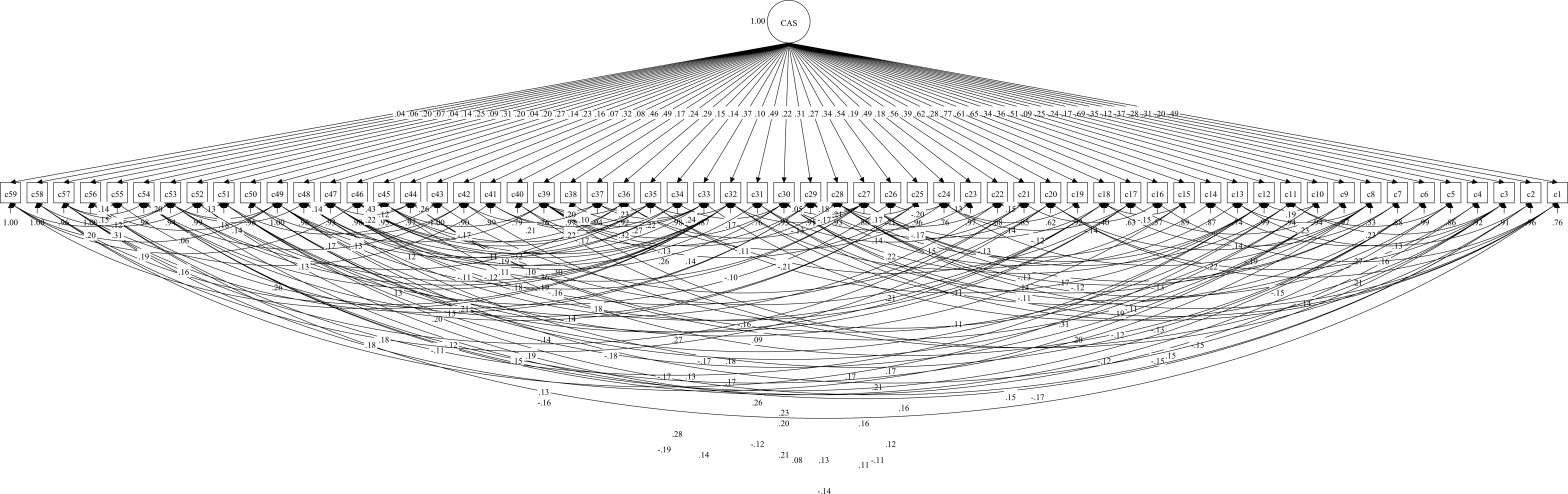
Model for confirmatory factor analysis (CFA).

The structure, which consists of 59 items and a single dimension identified through EFA, was tested utilizing CFA. MPlus 7.4 was used to perform CFA of the CAS, with the maximum likelihood method chosen for estimation of the model parameters. Based on established guidelines for goodness of fit, the model was considered acceptable. The CFI and TLI values were both above 0.90, while the RMSEA and SRMR values were below 0.08, aligning with the recommended cutoffs (CFI = 0.91, TLI = 0.91, RMSEA = 0.02, SRMR = 0.06). The model for CAS is illustrated in **Figure 2**.

Applying the modification index (MI) to address model misspecification (22), error covariances were added between variables, as shown in **Figure 2**, resulting in acceptable fit indices and a good model fit. The addition of these error covariances was guided by theoretical and conceptual considerations rather than statistical criteria alone. Specifically, correlated errors were allowed between items that share similar contents, wording, or closely related aspects of childhood adversity. For example, items related to parental neglect (e.g., lack of attention or unmet needs), emotional abuse (e.g., criticism or humiliation), and family dysfunction (e.g., parental conflict or violence) are conceptually related and may share variance beyond the general factor. Therefore, allowing correlations between their error terms was considered theoretically meaningful and consistent with the underlying construct of childhood adversity.

**Table 2** shows that the factor loadings ranged from 0.04 (item 55) to 0.77 (item 18) in the model. Of the 59 items, the factor loadings of 10 items were not statistically significant (items 12, 32, 41, 43, 49, 52, 55, 56, 58, and 59). However, none of these items were deleted from the model to preserve content validity and maintain acceptable levels of the goodness-of-fit indices. Moreover, the reliability coefficient of the scale was calculated as 0.99, indicating excellent reliability as it exceeds 0.90 (30). Item–rest correlation, a fundamental step in improving the validity and reliability of scale development, was also applied to assess the degree to which an individual item on the scale correlates with the total score of the remaining items on the scale (31). **Table 3** shows that the item–rest correlation values ranged from 0.66 (items 1 and 3) to 0.96 (item 32). The items with insignificant factor loadings nevertheless showed statistically significant item–rest correlations, ranging from 0.86 to 0.96.

**Table 2:** Factor loading values of the items.

Item	Factor loading value	Item	Factor loading value	Item	Factor loading value	Item	Factor loading value
Item 1	0.49	Item 16	0.65	Item 31	0.49	Item 46	0.14
Item 2	0.20	Item 17	0.61	Item 32	0.10	Item 47	0.27
Item 3	0.31	Item 18	0.77	Item 33	0.37	Item 48	0.20
Item 4	0.29	Item 19	0.28	Item 34	0.14	Item 49	0.04
Item 5	0.37	Item 20	0.62	Item 35	0.15	Item 50	0.20
Item 6	0.12	Item 21	0.39	Item 36	0.29	Item 51	0.31
Item 7	0.35	Item 22	0.56	Item 37	0.24	Item 52	0.09
Item 8	0.69	Item 23	0.18	Item 38	0.17	Item 53	0.25
Item 9	0.17	Item 24	0.49	Item 39	0.49	Item 54	0.14
Item 10	0.24	Item 25	0.19	Item 40	0.46	Item 55	0.04
Item 11	0.25	Item 26	0.54	Item 41	0.08	Item 56	0.07
Item 12	0.09	Item 27	0.34	Item 42	0.32	Item 57	0.20
Item 13	0.51	Item 28	0.27	Item 43	0.07	Item 58	0.06
Item 14	0.36	Item 29	0.31	Item 44	0.16	Item 59	0.05
Item 15	0.34	Item 30	0.22	Item 45	0.23		

**Table 3:** Item-rest correlations of the items.

Item	Item–rest correlation	Item	Item-rest correlation	Item	Item-rest correlation	Item	Item-rest correlation
Item 1	0.66	Item 16	0.88	Item 31	0.94	Item 46	0.94
Item 2	0.69	Item 17	0.89	Item 32	0.96	Item 47	0.93
Item 3	0.66	Item 18	0.89	Item 33	0.95	Item 48	0.95
Item 4	0.67	Item 19	0.9	Item 34	0.94	Item 49	0.95
Item 5	0.67	Item 20	0.89	Item 35	0.95	Item 50	0.94
Item 6	0.8	Item 21	0.92	Item 36	0.94	Item 51	0.92
Item 7	0.77	Item 22	0.92	Item 37	0.95	Item 52	0.93
Item 8	0.8	Item 23	0.94	Item 38	0.94	Item 53	0.93
Item 9	0.78	Item 24	0.92	Item 39	0.94	Item 54	0.93
Item 10	0.81	Item 25	0.94	Item 40	0.93	Item 55	0.93
Item 11	0.83	Item 26	0.93	Item 41	0.95	Item 56	0.91
Item 12	0.86	Item 27	0.94	Item 42	0.95	Item 57	0.92
Item 13	0.84	Item 28	0.95	Item 43	0.94	Item 58	0.91
Item 14	0.83	Item 29	0.94	Item 44	0.94	Item 59	0.91
Item 15	0.84	Item 30	0.72	Item 45	0.94		

### Convergent validity

To assess the convergent validity of the CAS, 125 adults aged 18 years and older living in Turkey completed both the CAS and the ACE questionnaire. A significant and positive correlation was found between the two measures (*r* = 0.73, *p* < 0.01), supporting the convergent validity of the CAS (32). While the strong correlation indicates that both tools measure similar constructs, the CAS offers a more detailed assessment of childhood adversity. In other words, CAS is more comprehensive than the short ACE questionnaire and measures differences in severity and frequency, which are not captured by ACE.

## Discussion

The aim of this study was to develop a comprehensive assessment tool that could validly and reliably measure adverse childhood experiences. Existing scales have generally failed to represent the full complexity of these experiences (33). This has been observed to create a gap in clinical practice and needs. For example, some of the available scales have failed to fully capture the profound impact of severe adverse childhood experiences, even though these experiences significantly contribute to mental health disorders such as PTSD, depression, anxiety, and DID.

A three-stage process of data collection was used to develop and validate this scale. The first phase utilized data from 338 participants to conduct an EFA. The results indicated that the first factor was roughly six times more influential than the second factor based on the eigenvalue and explained variance. Furthermore, more than half of the variance was explained by the first factor, indicating that the scale is unidimensional (27, 28). In the second stage, CFA was conducted with data from 413 participants. The model fit indices indicated an acceptable fit based on theoretically informed modifications.

The CTQ (9) and the LEQ (10) are the most widely used tools for assessing childhood maltreatment in adults. These instruments primarily focus on domains such as neglect and emotional, physical, and sexual abuse. However, years of experience working with clients suffering from childhood trauma have shown that other adversities that are not included in widely used scales, such as natural disasters, encountering menstrual blood, or witnessing the child’s parents having sex, could also cause trauma. Studies have consistently shown that exposure to such events is associated with increased rates of PTSD, depression, anxiety, and other mental health issues (34, 35). Traumatic experiences—such as seeing menstrual blood for the first time or witnessing parents having sex—can be seen as neglect and are usually included in some of the existing scales. However, these specific events may be missed due to the current tools not directly assessing them. After reviewing the files of 300 clients with his team, the first author—who has over 30 years of clinical experience, in particular with DID clients—discovered a need for a new scale that would comprehensively assess childhood adversities, especially for DID cases.

The CAS shows potential for use both in research and clinical settings. This scale offers concrete benefits, particularly in trauma-focused therapies. In order to identify particular traumatic events and adjust treatment plans accordingly, it can be utilized in therapeutic settings to assess individuals who have experienced severe childhood trauma. Moreover, it could help therapists identify key traumas when working with DID, where alternate identities often emerge in response to specific traumatic experiences. Identifying these traumas could also facilitate easier access to the alternate identities. Furthermore, in eye movement desensitization and reprocessing (EMDR) therapy, the scale could be helpful in identifying which memories need to be processed, which expedites the healing process.

Because the structure and cultural characteristics of each society differ, adversities and traumatic experiences could also vary. Therefore, it is important to have a scale developed in accordance with the unique characteristics of each society. Although this perspective is changing in the new generation, menstruation in Turkey is still often viewed as a negative and impure process, sometimes described as “being ill” (36). In this context, the fact that the CAS scale takes into account such culturally specific situations can be considered one of its strengths. This scale is also believed to be valuable for anyone working in the field of trauma internationally, as it is based on the traumatic experiences of individuals in a clinical sample. With cultural adaptation studies, it could become an international scale that contributes to different cultures as well.

The CAS scale was developed and validated in Turkish. When translating the list of adverse experiences into English, the translation and back-translation processes were not carried out in accordance with the scale adaptation guidelines. The original Turkish scale and the list of adverse experiences in English are included in **Appendix 2**.

Future studies should adapt the scale to different cultures in order to ensure broader international applicability. Furthermore, future studies should also examine measurement invariance to ensure conceptual and psychometric equivalence of the scale across various populations.

Unlike the CTQ and LEQ, which include multiple subscales, the CAS scale developed in this study is unidimensional. Unidimensional measures may offer practical advantages in terms of ease of administration and interpretation, particularly in applied settings (37). Furthermore, unidimensional measurements generally yield higher reliability results because they focus on a single construct (38).

However, childhood adverse experiences are complex and multidimensional. Therefore, a unidimensional structure may not fully reflect the different types and characteristics of adverse experiences (1). Future studies may therefore develop shorter forms of the scale to improve feasibility, applying alternative models such as multidimensional or bifactor structures.

This scale encompasses negative consequences that can lead to various traumatic outcomes. Some of these negative consequences may seem insignificant or meaningless to many people; however, each item, developed through clinical studies, has the potential to raise awareness and improve sensitivity among parents and the wider community. In this regard, the scale could also be valuable for those developing awareness-raising and prevention programs, helping to address and mitigate the long-term impacts of these adversities.

Furthermore, while previous studies have generally focused on the frequency of traumatic experiences, CAS includes variables such as coping with trauma, whether the individual was directly affected by the negative event, their age at the time, and how impactful it was. By capturing critical contextual information, it may offer clinicians a more comprehensive understanding of each individual’s negative childhood experiences.

Similarly, the question of how early life experiences are remembered could have been added to the CAS. However, the inclusion of such a component would likely increase the complexity of the scale. It may bring up the issue of whether the person remembers “through their own memories” or “through the influence of others” (such as parents or grandparents). Over-reliance on self-reports is a significant shortcoming of trauma research. Frissa et al. (39) noted that recall bias is a well-documented phenomenon, as people may struggle to recall their early-life traumas.

While developed to address a clinical need, the CAS also has some limitations. For example, the length of the CAS might make it difficult to use and not suitable for community screening. Furthermore, this study showed that a number of items had low factor loadings. This could mean that these items may be less strongly related to the main construct. However, these items were retained as they represent significant dimensions of childhood adversity. The high item–total correlations, in particular, support this contribution. Moreover, the scale is applicable to Turkish communities as it was created using client files from a Turkish sample. In addition, data collection during the scale development phase utilized a convenience sampling method. This sampling method may limit the generalizability of the findings. Therefore, future studies should be conducted with participants from different sociodemographic and clinical backgrounds selected using probability-based sampling methods. This will increase the external validity of the scale. Finally, the online collection of data during the scale development phase may have introduced potential biases. Individuals with greater Internet access and higher digital literacy may have been particularly likely to participate in this study. Future research could combine online and in-person approaches to reduce sampling bias and increase representativeness.

In summary, the CAS, which possesses good psychometric properties, is a useful tool for both research and clinical use. By capturing important aspects of childhood adversity, it may contribute to both research and clinical assessment. However, testing the scale in diverse samples will enhance its validity. Specifically, future studies should examine the use of the CAS in different cultures and non-clinical populations and also investigate its ability to predict psychological outcomes. This will increase the usefulness of the CAS in both research and clinical settings.

## Data Availability

The raw data supporting the conclusions of this article will be made available by the authors, without undue reservation.
