# Supplementary material for: Development and validation of a measure of early adverse experiences: childhood adversity scale
Source: Front Psychiatry. 2026 May 12;17:1701294. doi: 10.3389/fpsyt.2026.1701294 (PMC13201247; doi:10.3389/fpsyt.2026.1701294)
Supplement: Supplementary file 1 [file SupplementaryFile1.docx]

**Appendix 1**

ÇOCUKLUK ÇAĞI OLUMSUZ YAŞANTILAR ÖLÇEĞİ (ÇOYÖ)

Aşağıda çocukluk çağında yaşanabilecek travmatik yaşantılar ile ilgili sorular vardır. “Bu ifade benim için geçerli” sorusuna ‘Hayır’ cevabını veren kişilerin bir sonraki soruya geçmeleri gerekmektedir.

Cevabınız ‘Evet ise soruları cevaplandırırken sizin için uygun olan rakamı **daire içerisine** alınız. “Bu olayı ne sıklıkta yaşadınız?” sorusuna yanıt verirken dikkat ediniz;

**1 = Çok Nadir**

**2 = Zaman Zaman**

**3 = Sıklıkla**

**4 = Çok Sık**

**5 = Her Zaman**

“Bu olay hangi çocukluk dönem ya da dönemlerinizde yaşandı?” sorusuna yanıt verirken dikkat ediniz;

**0-1 Yaş = Bebeklik**

**1-3 Yaş = Küçük Çocukluk**

**3-6 Yaş = Erken Çocukluk-Okul Öncesi**

**6-9 Yaş = Erken Çocukluk**

**9-11 Yaş= Orta Çocukluk**

“Bu olay sizi ne kadar etkiledi?” sorusuna yanıt verirken dikkat ediniz;

**1. = Hiç Etkilemedi**

**2. = Biraz Etkiledi**

**3. = Orta Derecede Etkiledi**

**4. = Çok Etkiledi**

**5. = İleri Derecede Etkiledi**

**Tarih:**

**Ad ve Soyad:**

| Olumsuz Yaşantılar | Bu İfade Benim  için Geçerli | Bu Olayı Ne Sıklıkta  Yaşadınız? | Bu Olay Hangi Çocukluk Dönem ya da  Dönemlerinizde Yaşandı? | Bu Olay Sizi Ne  Kadar Etkiledi? |
| --- | --- | --- | --- | --- |
| 1.Çocukluğumda ebeveynlerim tarafından başka çocuklarla  kıyaslandım. | Evet Hayır 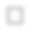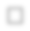 | **1** \| **2** \| **3** \| **4** \| **5** | **0-1 Yaş**\|**1-3 Yaş**\|**3-6 Yaş**\|**6-9 Yaş**\|**9-11 Yaş** | **1** \| **2** \| **3** \| **4** \| **5** |
| 2.Çocukluğumda ailem tarafından  cezalandırılmak adına kapalı yerlere kilitli bırakıldım. | Evet Hayır 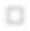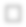 | **1** \| **2** \| **3** \| **4** \| **5** | **0-1 Yaş**\|**1-3 Yaş**\|**3-6 Yaş**\|**6-9 Yaş**\|**9-11 Yaş** | **1** \| **2** \| **3** \| **4** \| **5** |
| 3.Çocukluğumda okulda tekrarlayıcı bir şekilde akran zorbalığına maruz kaldım (Ad takılma, gruptan  dışlanma, eşyalarını zorla alma, alay  edilme vb). | Evet Hayır 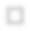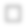 | **1** \| **2** \| **3** \| **4** \| **5** | **0-1 Yaş**\|**1-3 Yaş**\|**3-6 Yaş**\|**6-9 Yaş**\|**9-11 Yaş** | **1** \| **2** \| **3** \| **4** \| **5** |
| 4.Çocukluğumda altımı ıslattığım için cezalandırıldım / azarlandım / dalga  geçildim. | Evet Hayır 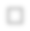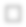 | **1** \| **2** \| **3** \| **4** \| **5** | **0-1 Yaş**\|**1-3 Yaş**\|**3-6 Yaş**\|**6-9 Yaş**\|**9-11 Yaş** | **1 \| 2** \| **3** \| **4** \| **5** |

| Olumsuz Yaşantılar | Bu İfade Benim  için Geçerli | Bu Olayı Ne Sıklıkta  Yaşadınız? | Bu Olay Hangi Çocukluk Dönem ya da  Dönemlerinizde Yaşandı? | Bu Olay Sizi Ne  Kadar Etkiledi? |
| --- | --- | --- | --- | --- |
| 5.Çocukken benden büyük  kardeşlerim / kuzenlerim bana sürekli kötü davranırdı (Alay etmek, ad  takmak, dışlanmak, dövmek vb.). | Evet Hayır 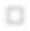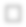 | **1** \| **2** \| **3** \| **4** \| **5** | **0-1 Yaş**\|**1-3 Yaş**\|**3-6 Yaş**\|**6-9 Yaş**\|**9-11 Yaş** | **1** \| **2** \| **3** \| **4** \| **5** |
| 6.Çocukluğumda kekelediğim için cezalandırıldım / azarlandım / dalga  geçildim. | Evet Hayır 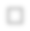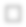 | **1** \| **2** \| **3** \| **4** \| **5** | **0-1 Yaş**\|**1-3 Yaş**\|**3-6 Yaş**\|**6-9 Yaş**\|**9-11 Yaş** | **1** \| **2** \| **3** \| **4** \| **5** |
| 7.Çocukluğumda dış dünyanın güvensiz olduğuna dair sürekli  korkutuldum, diğer çocuklar dışarıda oyun oynarken ben sokağa  çıkarılmayıp evde tutuldum. | Evet Hayır 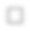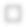 | **1** \| **2** \| **3** \| **4** \| **5** | **0-1 Yaş**\|**1-3 Yaş**\|**3-6 Yaş**\|**6-9 Yaş**\|**9-11 Yaş** | **1** \| **2** \| **3** \| **4** \| **5** |
| 8.Çocukluğumda annemin / babamın öfkelenmesinden bana kötü  davranabilecekleri için korkardım. | Evet Hayır 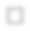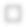 | **1** \| **2** \| **3** \| **4** \| **5** | **0-1 Yaş**\|**1-3 Yaş**\|**3-6 Yaş**\|**6-9 Yaş**\|**9-11 Yaş** | **1** \| **2** \| **3** \| **4** \| **5** |
| 9.Çocukluğumda tekrarlayıcı bir şekilde korku filmleri / vahşet  sahneleri içeren filmler seyrettim. | Evet Hayır 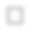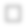 | **1** \| **2** \| **3** \| **4** \| **5** | **0-1 Yaş**\|**1-3 Yaş**\|**3-6 Yaş**\|**6-9 Yaş**\|**9-11 Yaş** | **1** \| **2** \| **3** \| **4** \| **5** |
| 10.Çocukluğumda (benden en az 4 yaş büyük kişiden/lerden) cinsel  tacize uğradım. | Evet Hayır 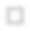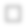 | **1** \| **2** \| **3** \| **4** \| **5** | **0-1 Yaş**\|**1-3 Yaş**\|**3-6 Yaş**\|**6-9 Yaş**\|**9-11 Yaş** | **1** \| **2** \| **3** \| **4** \| **5** |
| 11.İlkokul öğretmenim beni  korkutur/döver/ bana bağırırdı. | Evet Hayır 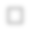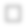 | **1** \| **2** \| **3** \| **4** \| **5** | **0-1 Yaş**\|**1-3 Yaş**\|**3-6 Yaş**\|**6-9 Yaş**\|**9-11**  **Yaş** | **1** \| **2** \| **3** \| **4** \| **5** |
| 12.Çocukluğumda benden büyüklerle  birlikte porno filmi izledim / izlettirildim. | Evet Hayır 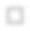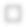 | **1** \| **2** \| **3** \| **4** \| **5** | **0-1 Yaş**\|**1-3 Yaş**\|**3-6 Yaş**\|**6-9 Yaş**\|**9-11 Yaş** | **1** \| **2** \| **3** \| **4** \| **5** |
| 13.Çocukluğumda tekrarlayıcı bir  şekilde dövüldüm. | Evet Hayır 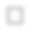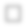 | **1** \| **2** \| **3** \| **4** \| **5** | **0-1 Yaş**\|**1-3 Yaş**\|**3-6 Yaş**\|**6-9 Yaş**\|**9-11**  **Yaş** | **1** \| **2** \| **3** \| **4** \| **5** |
| 14.Çocukluğumda farklı fiziksel  özelliklerimden dolayı (kilolu, zayıf, kısa,uzun, çilli vb) aşağılandım. | Evet Hayır 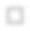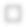 | **1** \| **2** \| **3** \| **4** \| **5** | **0-1 Yaş**\|**1-3 Yaş**\|**3-6 Yaş**\|**6-9 Yaş**\|**9-11 Yaş** | **1** \| **2** \| **3** \| **4** \| **5** |
| 15.Çocukluğumda kişisel eşyalarım  benden izin alınmadan / haberim olmadan çöpe atıldı. | Evet Hayır 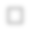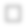 | **1** \| **2** \| **3** \| **4** \| **5** | **0-1 Yaş**\|**1-3 Yaş**\|**3-6 Yaş**\|**6-9 Yaş**\|**9-11 Yaş** | **1** \| **2** \| **3** \| **4** \| **5** |
| 16.Çocukluğumda annem / babam  tarafından sürekli aşağılandım / eleştirildim. | Evet Hayır 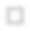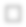 | **1** \| **2** \| **3** \| **4** \| **5** | **0-1 Yaş**\|**1-3 Yaş**\|**3-6 Yaş**\|**6-9 Yaş**\|**9-11 Yaş** | **1** \| **2** \| **3** \| **4** \| **5** |
| 17.Çocukluğumda yalnızlık içinde  büyüdüm. | Evet Hayır 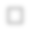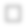 | **1** \| **2** \| **3** \| **4** \| **5** | **0-1 Yaş**\|**1-3 Yaş**\|**3-6 Yaş**\|**6-9 Yaş**\|**9-11**  **Yaş** | **1** \| **2** \| **3** \| **4** \| **5** |
| 18.Çocukken sevilmediğimi  hissederdim. | Evet Hayır 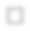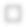 | **1** \| **2** \| **3** \| **4** \| **5** | **0-1 Yaş**\|**1-3 Yaş**\|**3-6 Yaş**\|**6-9 Yaş**\|**9-11**  **Yaş** | **1** \| **2** \| **3** \| **4** \| **5** |
| 19.Çocukluğumda annem / babam hasta olan / ölen kardeşimden sonra  beni ihmal etti / unuttu. | Evet Hayır 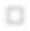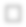 | **1** \| **2** \| **3** \| **4** \| **5** | **0-1 Yaş**\|**1-3 Yaş**\|**3-6 Yaş**\|**6-9 Yaş**\|**9-11 Yaş** | **1** \| **2** \| **3** \| **4** \| **5** |
| 20.Çocukluğumda annem bana yeteri kadar ilgi göstermedi ve ihtiyaçlarımı  karşılamadı. | Evet Hayır 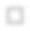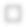 | **1** \| **2** \| **3** \| **4** \| **5** | **0-1 Yaş**\|**1-3 Yaş**\|**3-6 Yaş**\|**6-9 Yaş**\|**9-11 Yaş** | **1** \| **2** \| **3** \| **4** \| **5** |

| Olumsuz Yaşantılar | Bu İfade Benim  için Geçerli | Bu Olayı Ne Sıklıkta  Yaşadınız? | Bu Olay Hangi Çocukluk Dönem ya da  Dönemlerinizde Yaşandı? | Bu Olay Sizi Ne  Kadar Etkiledi? |
| --- | --- | --- | --- | --- |
| 21.Çocukluğumda annem / babam  benim yıl sonu gösterisi / spor /müzik  /tiyatro vb. aktivitelerimi seyretmeye gelmezdi. | Evet Hayır 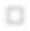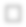 | **1** \| **2** \| **3** \| **4** \| **5** | **0-1 Yaş**\|**1-3 Yaş**\|**3-6 Yaş**\|**6-9 Yaş**\|**9-11 Yaş** | **1** \| **2** \| **3** \| **4** \| **5** |
| 22.Çocukluğumda annem / babam iyi  bir şey yapsam bile takdir etmezlerdi. | Evet Hayır 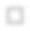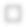 | **1** \| **2** \| **3** \| **4** \| **5** | **0-1 Yaş**\|**1-3 Yaş**\|**3-6 Yaş**\|**6-9 Yaş**\|**9-11**  **Yaş** | **1** \| **2** \| **3** \| **4** \| **5** |
| 23.Doğduğumda anne / babamın istediği cinsiyette olmadığım için  ilgisiz bırakıldım. | Evet Hayır 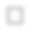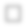 | **1** \| **2** \| **3** \| **4** \| **5** | **0-1 Yaş**\|**1-3 Yaş**\|**3-6 Yaş**\|**6-9 Yaş**\|**9-11 Yaş** | **1** \| **2** \| **3** \| **4** \| **5** |
| 24.Çocukluğumda annem / babam  kardeşimi bana tercih eder kayırırdı. | Evet Hayır 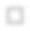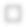 | **1** \| **2** \| **3** \| **4** \| **5** | **0-1 Yaş**\|**1-3 Yaş**\|**3-6 Yaş**\|**6-9 Yaş**\|**9-11**  **Yaş** | **1** \| **2** \| **3** \| **4** \| **5** |
| 25.Çocukluğumda diş problemi, görme sorunu, şaşılık vb. fiziksel  sağlık problemim olmasına rağmen,  ailem tedavimi yaptırmadı. | Evet Hayır 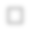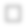 | **1** \| **2** \| **3** \| **4** \| **5** | **0-1 Yaş**\|**1-3 Yaş**\|**3-6 Yaş**\|**6-9 Yaş**\|**9-11 Yaş** | **1** \| **2** \| **3** \| **4** \| **5** |
| 26.Çocukluğumda kaygı, depresyon, tik vb. ruhsal sorunlar yaşamama  rağmen ailem bunlar için çözüm  aramadı. | Evet Hayır 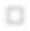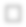 | **1** \| **2** \| **3** \| **4** \| **5** | **0-1 Yaş**\|**1-3 Yaş**\|**3-6 Yaş**\|**6-9 Yaş**\|**9-11 Yaş** | **1** \| **2** \| **3** \| **4** \| **5** |
| 27.Çocukluğumda annem / babam  beni evde yalnız bırakarak dışarı çıkardı. | Evet Hayır 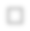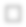 | **1** \| **2** \| **3** \| **4** \| **5** | **0-1 Yaş**\|**1-3 Yaş**\|**3-6 Yaş**\|**6-9 Yaş**\|**9-11 Yaş** | **1** \| **2** \| **3** \| **4** \| **5** |
| 28. Çocukluğumda aç  kalırdım/bırakılırdım. | Evet Hayır 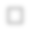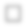 | **1** \| **2** \| **3** \| **4** \| **5** | **0-1 Yaş**\|**1-3 Yaş**\|**3-6 Yaş**\|**6-9 Yaş**\|**9-11**  **Yaş** | **1** \| **2** \| **3** \| **4** \| **5** |
| 29.Çocukluğum boyunca evde  hizmetçi gibi çalıştırıldım / annem babam yerine kardeşlerime bakmak zorunda kaldım /  büyüklerin yapmaları gereken işler  bana yaptırıldı. | Evet Hayır 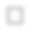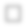 | **1** \| **2** \| **3** \| **4** \| **5** | **0-1 Yaş**\|**1-3 Yaş**\|**3-6 Yaş**\|**6-9 Yaş**\|**9-11 Yaş** | **1** \| **2** \| **3** \| **4** \| **5** |
| 30.Çocukluğumda bana adet kanaması önceden anlatılmadığı için  ilk adet kanamamda çok korktum. | Evet Hayır 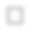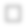 | **1** \| **2** \| **3** \| **4** \| **5** | **0-1 Yaş**\|**1-3 Yaş**\|**3-6 Yaş**\|**6-9 Yaş**\|**9-11 Yaş** | **1** \| **2** \| **3** \| **4** \| **5** |
| 31.Çocukluğumda annemden /  babamdan aşırı korkar onun yanında rahat olamaz, onunla konuşmaya  dahi cesaret edemezdim. | Evet Hayır 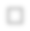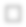 | **1** \| **2** \| **3** \| **4** \| **5** | **0-1 Yaş**\|**1-3 Yaş**\|**3-6 Yaş**\|**6-9 Yaş**\|**9-11 Yaş** | **1** \| **2** \| **3** \| **4** \| **5** |
| 32.Çocukken bir başkasına evlatlık  verildim. | Evet Hayır 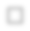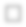 | **1** \| **2** \| **3** \| **4** \| **5** | **0-1 Yaş**\|**1-3 Yaş**\|**3-6 Yaş**\|**6-9 Yaş**\|**9-11**  **Yaş** | **1** \| **2** \| **3** \| **4** \| **5** |
| 33.Çocukluğumda babamın anneme  şiddet uygulamalarına şahit oldum. | Evet Hayır 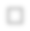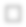 | **1** \| **2** \| **3** \| **4** \| **5** | **0-1 Yaş**\|**1-3 Yaş**\|**3-6 Yaş**\|**6-9 Yaş**\|**9-11**  **Yaş** | **1** \| **2** \| **3** \| **4** \| **5** |
| 34.Çocukluğumda annem / babam / kardeşlerim alkolik / uyuşturucu  madde bağımlısıydı. | Evet Hayır 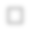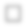 | **1** \| **2** \| **3** \| **4** \| **5** | **0-1 Yaş**\|**1-3 Yaş**\|**3-6 Yaş**\|**6-9 Yaş**\|**9-11 Yaş** | **1** \| **2** \| **3** \| **4** \| **5** |

| Olumsuz Yaşantılar | Bu İfade Benim  için Geçerli | Bu Olayı Ne Sıklıkta  Yaşadınız? | Bu Olay Hangi Çocukluk Dönem ya da  Dönemlerinizde Yaşandı? | Bu Olay Sizi Ne  Kadar Etkiledi? |
| --- | --- | --- | --- | --- |
| 35.Anne-babam boşandı/ ikisinden biriyle veya ikisi ile ilişkim koptu /  bariz bir şekilde azaldı. | Evet Hayır 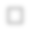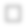 | **1** \| **2** \| **3** \| **4** \| **5** | **0-1 Yaş**\|**1-3 Yaş**\|**3-6 Yaş**\|**6-9 Yaş**\|**9-11 Yaş** | **1** \| **2** \| **3** \| **4** \| **5** |
| 36.Çocukluğumda annemin /  babamın şizofreni, bipolar, obsesif  kompulsif, depresyon vb. ruhsal  rahatsızlıkları vardı. | Evet Hayır 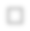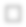 | **1** \| **2** \| **3** \| **4** \| **5** | **0-1 Yaş**\|**1-3 Yaş**\|**3-6 Yaş**\|**6-9 Yaş**\|**9-11 Yaş** | **1** \| **2** \| **3** \| **4** \| **5** |
| 37. Çocukluğumda ebeveynlerimden birinin diğerini aldatmasına şahit  oldum. | Evet Hayır 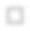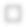 | **1** \| **2** \| **3** \| **4** \| **5** | **0-1 Yaş**\|**1-3 Yaş**\|**3-6 Yaş**\|**6-9 Yaş**\|**9-11 Yaş** | **1** \| **2** \| **3** \| **4** \| **5** |
| 38. Çocukluğumda evde  oynayabileceğim oyuncaklarım yoktu. | Evet Hayır 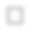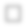 | **1** \| **2** \| **3** \| **4** \| **5** | **0-1 Yaş**\|**1-3 Yaş**\|**3-6 Yaş**\|**6-9 Yaş**\|**9-11**  **Yaş** | **1** \| **2** \| **3** \| **4** \| **5** |
| 39. Çocukluğumda anne ve babamın  tekrarlayıcı kavgaları arasında kaldım. | Evet Hayır 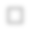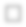 | **1** \| **2** \| **3** \| **4** \| **5** | **0-1 Yaş**\|**1-3 Yaş**\|**3-6 Yaş**\|**6-9 Yaş**\|**9-11**  **Yaş** | **1** \| **2** \| **3** \| **4** \| **5** |
| 40.Çocukken, annemin / babamın aile büyükleri tarafından ezilmesine /  aşağılanmasına şahitlik ettim. | Evet Hayır 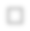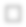 | **1** \| **2** \| **3** \| **4** \| **5** | **0-1 Yaş**\|**1-3 Yaş**\|**3-6 Yaş**\|**6-9 Yaş**\|**9-11 Yaş** | **1** \| **2** \| **3** \| **4** \| **5** |
| 41.Çocukluğumda annem / babam /  kardeşim hapishaneye girdi. | Evet Hayır 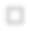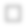 | **1** \| **2** \| **3** \| **4** \| **5** | **0-1 Yaş**\|**1-3 Yaş**\|**3-6 Yaş**\|**6-9 Yaş**\|**9-11**  **Yaş** | **1** \| **2** \| **3** \| **4** \| **5** |
| 42.Çocukken anne ve babamın cinsel  ilişkisine şahit oldum. | Evet Hayır 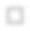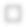 | **1** \| **2** \| **3** \| **4** \| **5** | **0-1 Yaş**\|**1-3 Yaş**\|**3-6 Yaş**\|**6-9 Yaş**\|**9-11**  **Yaş** | **1** \| **2** \| **3** \| **4** \| **5** |
| 43.Çocukken sık taşındım ve/veya sık  okul değiştirdim. | Evet Hayır 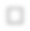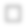 | **1** \| **2** \| **3** \| **4** \| **5** | **0-1 Yaş**\|**1-3 Yaş**\|**3-6 Yaş**\|**6-9 Yaş**\|**9-11**  **Yaş** | **1** \| **2** \| **3** \| **4** \| **5** |
| 44.Çocukluğumda anne veya babam iş, sağlık vb. sebeplerle evden uzun  süre uzak kalırdı. | Evet Hayır 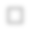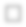 | **1** \| **2** \| **3** \| **4** \| **5** | **0-1 Yaş**\|**1-3 Yaş**\|**3-6 Yaş**\|**6-9 Yaş**\|**9-11 Yaş** | **1** \| **2** \| **3** \| **4** \| **5** |
| 45.Çocukken anne babamdan uzak bir yerde büyütüldüğüm dönem/ler  oldu. | Evet Hayır 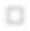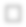 | **1** \| **2** \| **3** \| **4** \| **5** | **0-1 Yaş**\|**1-3 Yaş**\|**3-6 Yaş**\|**6-9 Yaş**\|**9-11 Yaş** | **1** \| **2** \| **3** \| **4** \| **5** |
| 46.Çocukken beni annem babam  yerine anneannem / babaannem  büyüttü. | Evet Hayır 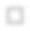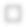 | **1** \| **2** \| **3** \| **4** \| **5** | **0-1 Yaş**\|**1-3 Yaş**\|**3-6 Yaş**\|**6-9 Yaş**\|**9-11 Yaş** | **1** \| **2** \| **3** \| **4** \| **5** |
| 47.Çocukluğumda annem / babam aşırı çalışır ve benimle vakit  geçirmezdi. | Evet Hayır 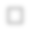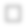 | **1** \| **2** \| **3** \| **4** \| **5** | **0-1 Yaş**\|**1-3 Yaş**\|**3-6 Yaş**\|**6-9 Yaş**\|**9-11 Yaş** | **1** \| **2** \| **3** \| **4** \| **5** |
| 48.Çocukluğumda yüksekten düşüp  zarar gördüm. | Evet Hayır 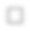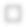 | **1** \| **2** \| **3** \| **4** \| **5** | **0-1 Yaş**\|**1-3 Yaş**\|**3-6 Yaş**\|**6-9 Yaş**\|**9-11**  **Yaş** | **1** \| **2** \| **3** \| **4** \| **5** |
| 49.Çocukluğumda asansörde tek  başıma kaldım. | Evet Hayır 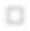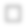 | **1** \| **2** \| **3** \| **4** \| **5** | **0-1 Yaş**\|**1-3 Yaş**\|**3-6 Yaş**\|**6-9 Yaş**\|**9-11**  **Yaş** | **1** \| **2** \| **3** \| **4** \| **5** |
| 50.Çocukluğumda diğer çocuklarla  cinsel içerikli oyunlar oynardım. | Evet Hayır 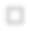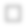 | **1** \| **2** \| **3** \| **4** \| **5** | **0-1 Yaş**\|**1-3 Yaş**\|**3-6 Yaş**\|**6-9 Yaş**\|**9-11**  **Yaş** | **1** \| **2** \| **3** \| **4** \| **5** |
| 51.Çocukluğumda yanımda cinlerle ilgili konuşulurdu veya cinci hocalara  götürülürdüm. | Evet Hayır | **1** \| **2** \| **3** \| **4** \| **5** | **0-1 Yaş**\|**1-3 Yaş**\|**3-6 Yaş**\|**6-9 Yaş**\|**9-11 Yaş** | **1** \| **2** \| **3** \| **4** \| **5** |
| 52.Çocukken ölmüş bir kişinin  bedenini gördüm. | Evet Hayır | **1** \| **2** \| **3** \| **4** \| **5** | **0-1 Yaş**\|**1-3 Yaş**\|**3-6 Yaş**\|**6-9 Yaş**\|**9-11**  **Yaş** | **1** \| **2** \| **3** \| **4** \| **5** |

| Olumsuz Yaşantılar | Bu İfade Benim  için Geçerli | Bu Olayı Ne Sıklıkta  Yaşadınız? | Bu Olay Hangi Çocukluk Dönem ya da  Dönemlerinizde Yaşandı? | Bu Olay Sizi Ne  Kadar Etkiledi? |
| --- | --- | --- | --- | --- |
| 53.Çocukken dışarıda kayboldum. | Evet Hayır | **1** \| **2** \| **3** \| **4** \| **5** | **0-1 Yaş**\|**1-3 Yaş**\|**3-6 Yaş**\|**6-9 Yaş**\|**9-11**  **Yaş** | **1** \| **2** \| **3** \| **4** \| **5** |
| 54.Çocukluğumda birileri üzerimdeki  değerli takıyı / paramı / eşyamı zorla almaya çalıştı. | Evet Hayır | **1** \| **2** \| **3** \| **4** \| **5** | **0-1 Yaş**\|**1-3 Yaş**\|**3-6 Yaş**\|**6-9 Yaş**\|**9-11 Yaş** | **1** \| **2** \| **3** \| **4** \| **5** |
| 55.Çocukluğumda benim / ailemin  zarar gördüğü trafik kazası geçirdim. | Evet Hayır | **1** \| **2** \| **3** \| **4** \| **5** | **0-1 Yaş**\|**1-3 Yaş**\|**3-6 Yaş**\|**6-9 Yaş**\|**9-11**  **Yaş** | **1** \| **2** \| **3** \| **4** \| **5** |
| 56.Çocukluğumda kalp, böbrek, kanser, yarık damak ve dudak vb uzun süreli sağlık sorunlarımdan  dolayı hastanede uzun süreli tedavi  gördüm. | Evet Hayır | **1** \| **2** \| **3** \| **4** \| **5** | **0-1 Yaş**\|**1-3 Yaş**\|**3-6 Yaş**\|**6-9 Yaş**\|**9-11 Yaş** | **1** \| **2** \| **3** \| **4** \| **5** |
| 57.Çocukluğumda bomba patlaması, silahlı çatışma veya kanlı kavgaya  şahit oldum. | Evet Hayır | **1** \| **2** \| **3** \| **4** \| **5** | **0-1 Yaş**\|**1-3 Yaş**\|**3-6 Yaş**\|**6-9 Yaş**\|**9-11 Yaş** | **1** \| **2** \| **3** \| **4** \| **5** |
| 58.Çocukluğumda ailemden biri /  yakın bir arkadaşım öldü. | Evet Hayır | **1** \| **2** \| **3** \| **4** \| **5** | **0-1 Yaş**\|**1-3 Yaş**\|**3-6 Yaş**\|**6-9 Yaş**\|**9-11**  **Yaş** | **1** \| **2** \| **3** \| **4** \| **5** |
| 59.Çocukluğumda deprem / sel /  yangın / salgın vb. doğa olaylarına maruz kaldım. | Evet Hayır | **1** \| **2** \| **3** \| **4** \| **5** | **0-1 Yaş**\|**1-3 Yaş**\|**3-6 Yaş**\|**6-9 Yaş**\|**9-11 Yaş** | **1** \| **2** \| **3** \| **4** \| **5** |

**Appendix 2: List of Adverse Experiences in CAS Scale**

| 1 | My parents compared me to other children in my childhood. |
| --- | --- |
| 2 | My family locked me in a room as a form of punishment in my childhood. |
| 3 | I was repeatedly subjected to peer bullying at school in my childhood (name- calling, exclusion from the group, having my belongings taken by force, being  mocked, etc.). |
| 4 | I was punished / scolded / teased for wetting myself in my childhood. |
| 5 | My older siblings / cousins constantly treated me badly in my childhood (teasing, name-calling, exclusion, hitting, etc.). |
| 6 | I was punished / scolded / teased for stuttering in my childhood. |
| 7 | I was constantly made to fear that the outside world was unsafe; whilst other children played outside, I was kept indoors and not allowed out onto the street in my childhood. |
| 8 | I was afraid that my mother / father might treat me badly when they got angry in  my childhood. |
| 9 | I repeatedly watched films containing horror scenes / scenes of violence in my childhood. |
| 10 | I was sexually abused by someone (or people) at least four years older than me in  my childhood. |
| 11 | My primary school teacher would scare me, hit me or shout at me. |
| 12 | I watched or was made to watch a pornographic film with people who are older than me in my childhood. |
| 13 | I was repeatedly beaten in my childhood. |
| 14 | I was humiliated because of my physical appearance (being overweight, underweight, short, tall, freckled, etc.) in my childhood. |
| 15 | My personal belongings were thrown away without my permission or knowledge  in my childhood. |
| 16 | I was constantly belittled or criticized by my mother or father in my childhood. |
| 17 | I grew up feeling lonely in my childhood. |
| 18 | I felt unloved in my childhood. |
| 19 | My mother/father neglected me or forgot about me after my sibling fell ill or died in my childhood. |
| 20 | My mother did not show me enough attention and did not meet my needs in my childhood. |
| 21 | My mother/father never came to watch my end-of-year show, sports, music,  theatre, or similar activities in my childhood. |
| 22 | My mother/father never praised me, even when I did something well in my childhood. |
| 23 | I was ignored in my childhood because I wasn’t the gender my mother/father had  wanted |
| 24 | My mother/father favored my sibling over me in my childhood. |

| 25 | Despite having physical health problems such as dental issues, vision problems, or strabismus, my family did not arrange my treatment in my childhood. |
| --- | --- |
| 26 | Despite experiencing mental health issues such as anxiety, depression, or tics in  my childhood, my family did not seek help for these. |
| 27 | My mother/father would leave me alone at home whilst they went out in my childhood. |
| 28 | I was left hungry in my childhood. |
| 29 | I was made to work like a servant at home in my childhood / I was forced to look  after my siblings instead of my parents / I was made to do the chores that adults should have done. |
| 30 | I was not told about menstruation (for women) / erections (for men) beforehand during my childhood, so I was very scared during my first experience. |
| 31 | I was terrified of my mother/father; I could not relax in their presence and did not  even dare to speak to them in my childhood. |
| 32 | I was given up for adoption to someone else in my childhood. |
| 33 | I witnessed my father being violent towards my mother in my childhood. |
| 34 | My mother / father / siblings were alcoholics / drug addicts in my childhood. |
| 35 | My parents divorced / my relationship with one or both broke down / became significantly strained in my childhood. |
| 36 | My mother / father suffered from mental health conditions such as schizophrenia,  bipolar disorder, obsessive-compulsive disorder, depression, etc. in my childhood. |
| 37 | I witnessed one of my parents cheating on the other in my childhood. |
| 38 | I had no toys to play with at home in my childhood. |
| 39 | I was caught in the middle of my parents’ repeated arguments in my childhood. |
| 40 | I witnessed my mother/father being bullied or humiliated by family elders in my childhood. |
| 41 | My mother / father / sibling went to prison in my childhood. |
| 42 | I witnessed my mother and father having sex in my childhood. |
| 43 | I moved house frequently and/or changed schools often in my childhood. |
| 44 | My mother or father would be away from home for long periods due to work, health, or other reasons in my childhood. |
| 45 | There were periods during my childhood when I was raised away from my  parents. |
| 46 | I was raised by my grandmother / grandfather instead of my mother and father in my childhood. |
| 47 | My mother / father worked excessively and did not spend time with me in my  childhood. |
| 48 | I fell from a height and was injured in my childhood. |
| 49 | I was left alone in a lift in my childhood. |
| 50 | I played games with other children that had sexual content in my childhood. |
| 51 | People would talk about jinn in my presence, or I was taken to jinn-exorcising clerics in my childhood. |
| 52 | I saw the body of a dead person in my childhood. |
| 53 | I got lost outside in my childhood. |

| 54 | Someone tried to forcibly take my valuable jewelry / money / belongings in my childhood. |
| --- | --- |
| 55 | I was involved in a traffic accident in which I or my family were injured in my  childhood. |
| 56 | I underwent long-term hospital treatment due to chronic health issues such as heart problems, kidney problems, cancer, cleft palate, and cleft lips, etc., in my childhood. |
| 57 | I witnessed a bomb explosion, an armed conflict, or a violent brawl in my childhood. |
| 58 | A member of my family or a close friend died in my childhood. |
| 59 | I was exposed to natural disasters such as earthquakes, floods, fires, or epidemics in my childhood. |
